# Methionine regulates self-renewal, pluripotency, and cell death of GIC through cholesterol—rRNA axis

**DOI:** 10.1186/s12885-022-10280-5

**Published:** 2022-12-23

**Authors:** Kiyotaka Yokogami, Taisei Kikuchi, Takashi Watanabe, Yasutaka Nakatake, Shinji Yamashita, Asako Mizuguchi, Hideo Takeshima

**Affiliations:** 1 Department of Neurosurgery, Faculty of Medicine, University of Miyazaki, Miyazaki, Japan; 2 Department of Infectious Diseases, Faculty of Medicine, University of Miyazaki, Miyazaki, Japan

**Keywords:** Methionine, Cholesterol biosynthesis, rRNA, ACA43, GIC

## Abstract

**Background:**

Glioma-initiating cells (GICs) are the source of glioma cells that can self-renew, have pluripotency, and are treatment-resistant, so are the starting point for relapse and eventual death despite multimodality therapy. L-[methyl-^11^C] methionine PET has observed high accumulation at the time of recurrence, it is important to understand the mechanism of tumor cell activation caused by the reorganization of methionine metabolism.

**Methods:**

We cultured cells in methionine-deprived culture medium for comprehensive analysis. Based on the obtained results, the possible target molecules were chemically inhibited and the respective markers were analyzed.

**Results:**

Methionine depletion markedly decreased proliferation and increased cell death of GICs. Decreased S-adenosyl-methionine, which is synthesized intracellularly by catalyzed by methionine adenosyltransferase using methionine, triggered the following: (i) global DNA demethylation, (ii) hyper-methylation of signaling pathways regulating pluripotency of stem cells, (iii) decreased expression of the core-genes and pluripotent markers of stem cells including *FOXM1, SOX2, SOX4, PROM1,* and *OLIG2*, (iv) decreased cholesterol synthesis and increased excretion mainly through decreased *SREBF2*, and (v) down-regulation of the large subunit of ribosomal protein configured 28S and *ACA43*, small nucleolar RNA guiding the pseudouridylation of 28S rRNA, which is essential for translation. In addition, inhibition of cholesterol synthesis with statin resulted in a phenotype similar to that of methionine depletion and decreases in stem cell markers and small nucleolar RNA *ACA43*. Moreover, suppression of *FOXM1* decreased stem cell markers such as *SOX4* and *PROM1*. The gene expression profile for cholesterol production was obtained from the Ivy Glioblastoma Atlas Project database and compared between tumor cells with relatively low methionine levels in areas of pseudopalisading arrangement around necrosis and tumor cells in the infiltrating region, showing that cells in the infiltrating region have higher capacity to produce cholesterol.

**Conclusions:**

Methionine metabolism is closely related with self-renewal, pluripotency, and cell death in GICs through modification of cholesterol biosynthesis, especially in the *SREBF2-FOXM1* and *ACA43* axis with modification of rRNA.

**Supplementary Information:**

The online version contains supplementary material available at 10.1186/s12885-022-10280-5.

## Introduction

Glioblastoma multiforme is the most common and deadly type of primary brain tumor with a median overall survival of less than 15 months despite aggressive treatment [1]. Glioblastoma multiforme can produce glioma cells and consequently self-renew, so glioma-initiating cells (GICs) are considered to be the root stem cell for tumorigenesis and recurrence. Cancer stem cells including GICs retain “plasticity” that changes dynamically as a result of metabolic reorganization.

Malignant transformation or recurrence of glioblastoma multiforme can be detected as increased methionine accumulation on PET before contrast enhancement is seen on MRI. Therefore, detailed understanding of the methionine metabolism of GICs may be important for tumor control. Methionine concentrations are expected to be high in areas of sparse tumor cells, but relatively low in areas of dense lesions, such as in the peri-necrotic pseudopalisading region, due to low blood supply. Therefore, glioblastoma multiforme may develop with nonuniform concentration of methionine within the same lesion. We hypothesized that reorganization of the methionine metabolism in the presence of restricted common source of methyl donors could modify the character of tumor cells as reflected by epigenetic status, and gene and protein expression profiles.

The present study showed that the methionine metabolism in GICs was closely related to cholesterol metabolism and rRNA expression, resulting in change in pluripotency, progression, and avoidance of cell death.

## Materials and methods

### Cell lines and culture conditions

Human GICs (MZGC1, MZGC2, MZGC3, MZGC4, MZGC5, MZGC6, MZGC7 and MZGC8 cells) were isolated and established as cell lines from the surgical tissues of patients treated in our institution from March 2015 to April 2021. GICs were cultured in Dulbecco’s modified Eagle’s medium/nutrient mixture F12 Ham’s liquid supplemented with SUBSER-ESrP® (Cell Science & Technology Institute, Inc., Sendai, Japan), CELRENA® medium (Cell Science & Technology Institute, Inc.), in 2-hydroxyethyl methacrylate -coated T75 flasks in a humidified incubator at 37 °C under an atmosphere of 5% CO_2_ and 95% air. For the methionine depletion studies, cells were plated on *laminin-coated 10-cm culture dishes* and cultured with complete CELRENA® medium or methionine deficient CELRENA® medium (custom medium CST1 MFG No. cn2243a0) for 48–72 h. Cells were dispersed onto laminin-coated 96-well plates, replaced with treated media on day 1, and time-lapse photography was performed with Incucyte® (Sartorius, Osaka, Japan). Simvastatin and FDI-6 were purchased from Selleck (Tokyo, Japan).

### Dye exclusion test

Dye exclusion testing was performed to analyze cell viability. Fixed amounts of cells were seeded and cultured in medium with (control) or without methionine for 3, 5 and 7 days. Harvested cells were suspended in phosphate buffered saline-containing trypan blue and then examined to determine the percentage of cells with blue clear cytoplasm (nonviable cells) versus total cells using a cell counter (LUNA, Logos Biosystems, Annandale, VA, USA). The relative rate against the control sample was calculated.

### Cell cycle analysis and detection of surface marker of GICs

Cells with or without treatment of methionine depletion/statin addition were collected using Accutase® solution (Sigma, USA). The Cell Cycle Phase Determination Kit (Cayman Chemical, Ann Arbor, MI, USA) was used for cell cycle analysis. In brief, collected cells were rinsed twice with buffer, then fixed at − 20 °C overnight. Cells were washed twice with ice-cold phosphate buffered saline, and stained with propidium iodide/RNase staining buffer solution in the dark for 30 min at room temperature. Then, cells were analyzed with a flow cytometer (Guava® EasyCyte™ Mini, Luminex Japan, Tokyo, Japan). A histogram of the cell cycle distribution was generated from 5000 events per sample and data were analyzed using Guava® Cell Cycle software. To detect CD133, the putative marker of GICs, by flow cytometry (FCM), 1 × 10^6^ cells derived from tumorspheres were analyzed using fluorescent (FITC) labelled antibodies (CD133/1 (AC133)-VioBright FITC, Miltenyi Biotec, Germany) and BD FACSCalibur (BD Biosciences, USA).

### RNA extraction including microRNA (miRNA), cDNA synthesis, and quantitative PCR (qPCR) analysis

RNA from cultured tumor cells was extracted with the *mir*Vana miRNA Isolation Kit (Ambion, Thermo Fisher Scientific K.K., Tokyo, Japan) or RNeasy Plus Mini Kit (QIAGEN, Germantown, MD, USA). For cDNA synthesis, RNA was reverse transcribed from random hexamers using the SuperScript™ VILO™ cDNA Synthesis Kit (Invitrogen, Life Technologies, Thermo Fisher Scientific, Waltham, MA, USA). Real-time qPCR was then performed in triplicate on the StepOne Plus or Quant3 (Applied Biosystems, Thermo Fisher Scientific) using SYBR™ Green Realtime PCR Master Mix (Applied Biosystems, Thermo Fisher Scientific) to determine the mRNA levels. PCR was performed using a 20 μl volume containing 2 µl cDNA, 300 µM of each primer, and 10 µl of 2 × PCR master mix under the following conditions: 95 °C for 10 min followed by 40 cycles of 95 °C for 15 s and annealing/extension at 60 °C for 1 min. The data were normalized to the amount of human *18S* rRNA, and the values are represented as the mean ± SD of 2^−ΔΔCt^ in a triplicate assay.

The primers used in this study were described in Supplementary Table S2.

### Colony formation assay

Cells were seeded into laminin-coated 12-well plates in triplicate at a density of 500 cells/well in 2 ml of medium with or without treatment medium. After 120 h, formed colonies were trypsinized, reseeded and cultured for further 72 h. After 192 h, the cell colonies were stained for 15 min with a solution containing 0.5% crystal violet and 25% methanol, followed by three rinses with tap water to remove excess dye. Each well was observed with Olympus IX71 microscope (Olympus, Tokyo, Japan).

### Gene and miRNA expression analysis

Gene and miRNA expression was analyzed with a GeneChip™ System with a Human Genome Clariom™ D Array and GeneChip™ miRNA 4.0 Array (Thermo Fisher Scientific) according to the manufacturer’s instructions. mRNA expression analysis used amplification and biotin labeling of fragmented cDNA with the biotin labeling system (GeneChip™ WT PLUS Reagent Kit, Thermo Fisher Scientific) in duplicate. miRNA expression analysis used biotin-labeled total RNA 1 µg including miRNA from tissue using the FlashTag™ Biotin HSR RNA Labeling Kit (Affymetrix®, Thermo Fisher Scientific). Labeled probes were hybridized to the Human Genome Clariom™ D Array (Thermo Fisher Scientific) or GeneChip™ miRNA 4.0 Array (Thermo Fisher Scientific) with the GeneChip™ Scanner 3000 7G (Affymetrix®, Thermo Fisher Scientific). Expression data of mRNA were extracted from image files using GeneSpring GX 14.9.1 (Agilent Technologies, Santa Clara, CA, USA). Normalization and expression value calculations were performed using the GeneSpring system. Signal values of miRNA were calculated using the Transcriptome Analysis Console software (Affymetrix®, Thermo Fisher Scientific). Statistical analysis was performed using two-tailed, unpaired t-tests. Any change ≥ twofold with *P* < 0.05 was considered statistically significant.

Enrichment analysis used Metascape (http://metascape.org/) and Gene Set Enrichment Analysis (GSEA) (http://www.broad.mit.edu/GSEA). Significance of biological processes was determined through *P*-values calculated on a hypergeometric distribution (log10). Metascape was used to conduct meta-analysis with generation of heat maps of gene ontology terms that hierarchically cluster together under experimental conditions. Statistical criteria for a differentially expressed gene included a change greater than 1.5 fold, and an overall false discovery rate smaller than 5% (change ≥  ± 1.5 fold and false discovery rate ≤ 0.05) with Benjamini-Hochberg correction (q) to account for multiple comparisons. GSEA using MSigDB (v7.4) was also used to determine the significance of a pre-defined gene set by comparing the correlation between expression and class distinction with other random situations. The significance threshold was set at nominal *P* value < 0.05 or false discovery rate q value < 0.25.

### Measurement of cholesterol, S-adenosyl-methionine (SAM), and S-adenosylhomocysteine (SAH) in tissue using ELISA

Measurement of cholesterol, SAM, and SAH used glioblastoma stem cells cultured with CELRENA medium or methionine-deprived CELRENA medium for 72 h and 10 × 10^6^ cells/cell pellets were homogenized by sonication in ice-cold phosphate buffered saline followed by centrifugation at 10,000 g for 15 min. Intracellular cholesterol, SAM, and SAH concentrations were measured using the Total Cholesterol Assay Kit (Colorimetric, Cell Biolabs, Inc., San Diego, CA, USA) and SAM and SAH ELISA Combo Kit (Cell Biolabs, Inc.) in accordance with the manufacturer's instructions under the indicated conditions. The standards were generated using the cholesterol standard and SAH/SAM-bovine serum albumin conjugate supplied in the kit. After the reaction was stopped, the OD450 and OD620 values were read using a SpectraMax iD3 Multi-Mode Microplate Reader (Molecular Devices, San Jose, CA, USA). SAH/SAM ratios were calculated as the ratio of SAM to average SAH using the intracellular concentration (µg/mL) to determine the relative methylation potential.

### Reduced Representation Bisulfite Sequencing (RRBS) and data analysis

RRBS libraries with single MspI digestion were constructed for glioblastoma stem cells. Briefly, 1 ug of genomic DNA was digested with 20U of MspI enzymes in 18 ul reaction mixtures at 37 °C for 2–3 h. After purification, the digested products were blunt-ended, and then dA added, followed by methylated-adapter ligation using a NEXTflex Bisulfite-Seq Ligation Kit. To obtain DNA fractions of > 100 bp, the MspI-digested products were purified using Agencourt AMPure XP (Beckman Coulter). Bisulfite conversion was conducted using an EX DNA Methylation Gold Kit following the manufacturer's instructions. The final libraries were generated by PCR amplification using NEXTflex Bisulfite-Seq Ligation Kit and sequenced using an Illumina HiSeqX to generate 150 bp pair-end reads (Supplementary Table S1).

Filtering sequence reads for both poor quality and adapters were performed via Trim Galore v0.6.4 (https://github.com/FelixKrueger/TrimGalore). Alignment to the human reference assembly hg19 and methylation calling was performed Bismark v0.21.0 (PMID:21,493,656).

CpG sites in the resulting RRBS data were then interrogated for methylation patterns and differential methylation (q value < 0.05 and methylation percentage difference of at least 25%) using the methylKit. The differential methylation data were then queried for differentially methylated regions (DMRs) using annotatr (https://bioconductor.org/packages/release/bioc/html/annotatr.html) and GeneDMRs, R package for Gene-based DMR analysis (https://www.biorxiv.org/content/10.1101/2020.04.11.037168v1.full). The resulting DMR outputs were also visualized in the genome browser or in the Integrative Genomics Viewer (IGV) program (https://software.broadinstitute.org/software/igv/). To establish the relationship between histone mark, insulators, transcription factors, and DMRs, the RRBS data conjunction with the data acquired from the Database of ChIP-Atlas Peak browser (https://chip-atlas.org/peak_browser) were projected on to the IGV.

### In silico analysis

Gene expression data obtained by the RNA sequencing technique involved in cholesterol metabolism in different anatomical regions (infiltrating tumor, pseudopalisading cells) of the glioblastoma multiform were identified using the Ivy Glioblastoma Atlas Project (Ivy GAP) database (http://glioblastoma.alleninstitute.org/).

### Statistical analysis

Statistical analyses were performed using GraphPad Prism 7 (GraphPad Software, La Jolla, CA, USA). Data are expressed as means ± SD of experiments performed in triplicate. One-way analysis of variance with Tukey’s post-hoc test or unpaired Student t-test was performed for comparisons of the quantitative data between groups. *P* < 0.05 was considered to indicate a statistically significant difference.

## Results

### Methionine depletion decreased cell proliferation and increased cell death

**Fig. 1:**
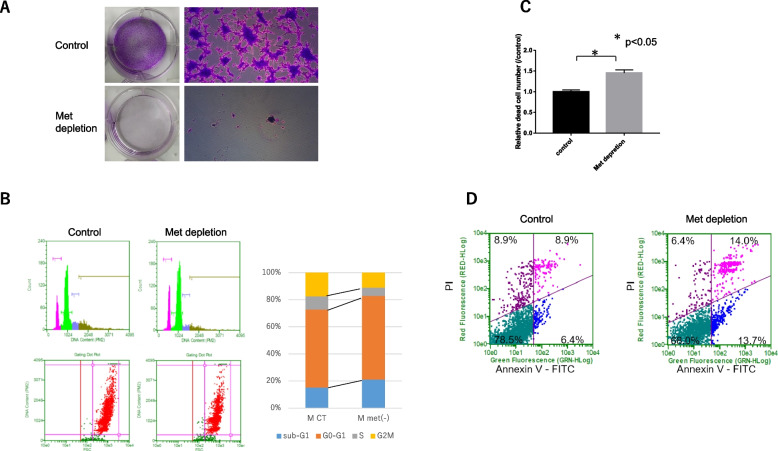
**A** Clonogenic assay: Methionine depletion for 5 days then reseeded for 3 days markedly decreased colony formation of MZGC1 cells. Quantitative analysis of colony areas in response to methionine depletion. **B** Fluorescence-activated cell sorter analysis to detect cell cycle distribution. Propidium iodide (PI) staining assay using a Cell Cycle Phase Determination Kit was performed on MZGC1 cells. Cell cycle analysis showed that methionine depletion for 3 days increased sub-G1 and decreased S, G2M populations. **C** Dye exclusion test showed that methionine depletion for 3 days increased dead cell rate. **D** Fluorescence-activated cell sorter analysis using annexin-V kit showed that methionine depletion induced apoptotic cell death

Clonogenic assay showed marked decreases in cell growth and sphere formation, suggesting reduced self-renewal capacity induced by the depletion of methionine (Fig. 1A). Fluorescence-activated cell sorter analysis and dye exclusion test were also performed to assess cell death or cell cycle arrest. Methionine depletion for 3 days resulted in increased sub-G1 population and cell death (Fig. 1B and C). Further fluorescence-activated cell sorter analysis using annexin-V showed apoptosis induced by methionine depletion, although the time to apoptosis varied from cell to cell (Fig. 1D, Supplementary Figure S1).

### Methionine depletion impaired SAM-dependent methyltransferase pathway

**Fig. 2:**
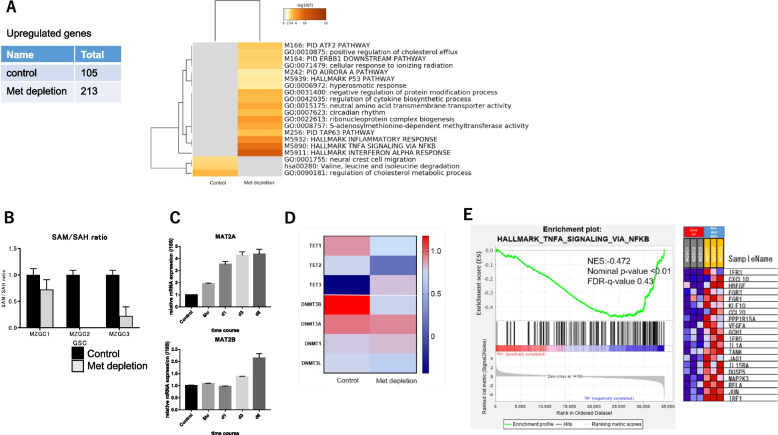
**A** Microarray analysis using Metascape. Heat map showing commonly altered pathways in the control and methionine-depleted states of three GICs (*MZGC1*, *MZGC2*, and *MZGC3*). Statistically enriched terms (gene ontology/Kyoto Encyclopedia of Genes and Genomes terms, canonical pathways, hall mark gene sets) are shown with their *P* value. The 20 families of pathways with the lowest -log10 adjusted *P* value are depicted. **B** Methionine depletion decreased (SAM/SAH ratio, which was measured using an ELISA Combo Kit in MZGC1 and MZGC2. **C** Methionine depletion increased *MAT2A* and *MAT2B* mRNA expression in time-dependent manners. Expression of MATII, alpha (*MAT2A*) and beta (MAT2B), was measured by qPCR after methionine depletion for 6 h, 24 h (day 1), 72 h (day 3), and 144 h (day 6) in MZGC3. **D** Heat map showing average interested gene mRNA expression level from expression array of MZGC1, MZGC2, and MZGC3 using Prism 7. Methionine depletion for 5 days downregulated *TET1, TET2, DNMT3A,* and *DNMT3B* mRNA expression. E: GSEA showing that methionine depletion significantly activated hallmark TNFA signaling via the NFKB pathway. GSEA of differential expression between control and methionine depletion states demonstrated that methionine-depleted GICs were enriched in hallmark TNFA signaling via the NFKB pathway. Heat map of top 20 genes from GSEA analysis is shown on the right. Red column corresponds to upregulated genes and blue column corresponds to downregulated genes

A comprehensive gene expression analysis was performed using a microarray to detect the characteristics of changes in specific molecular networks and canonical signaling pathways induced by modified methionine metabolism. A total of 213 genes were upregulated in the methionine withdrawal group, which were enriched in 17 Metascape terms/pathways, including 1) SAM-dependent methyltransferase pathway, 2) hallmark TNFA signaling via the nuclear factor-kappa B (NFKB), 3) regulation of cytokine biosynthetic process, 4) neutral amino acid transmembrane transporter activity, 5) circadian rhythm, 6) ribonucleoprotein complex biogenesis, 7) negative regulation of protein modification process, and 8) positive regulation of cholesterol efflux (Fig. 2A, Supplementary Figure S2). In contrast, 115 genes downregulated in the methionine withdrawal group were enriched in terms, regulation of cholesterol metabolic process and neural crest cell migration. SAM is a methionine derivative that is essential for the catalytic reaction of methylase. Intracellular SAM levels were reduced by methionine depletion (Fig. 2B). SAM is synthesized intracellularly by methionine adenosyltransferase (MAT) using the essential amino acids methionine and ATP as substrates. SAM is a methyl group donor and the dependent pathways include *MAT2A, MAT2B, DNMT1, DNMT3A,* and *DNMT3B*. qPCR and expression array were performed to examine whether methionine depletion in GICs could functionally regulate histone and DNA methylase. Methionine depletion up-regulated *MAT2A* and *MAT2B* in time-dependent manners (Fig. 2C). *DNMT3B*, *DNMT3A*, *TET1*, and *TET2* were down-regulated (Fig. 2D). As shown above, hallmark TNFA signaling via the NFKB is activated in the methionine-depleted state, as we confirmed in GSEA (Fig. 2E). Cell death via apoptosis might result from the activated TNFA signaling pathway (Figs. 1B–D, 2A, and E).

### Methionine depletion leads to pathway-dependent changes in methylation status

**Fig. 3:**
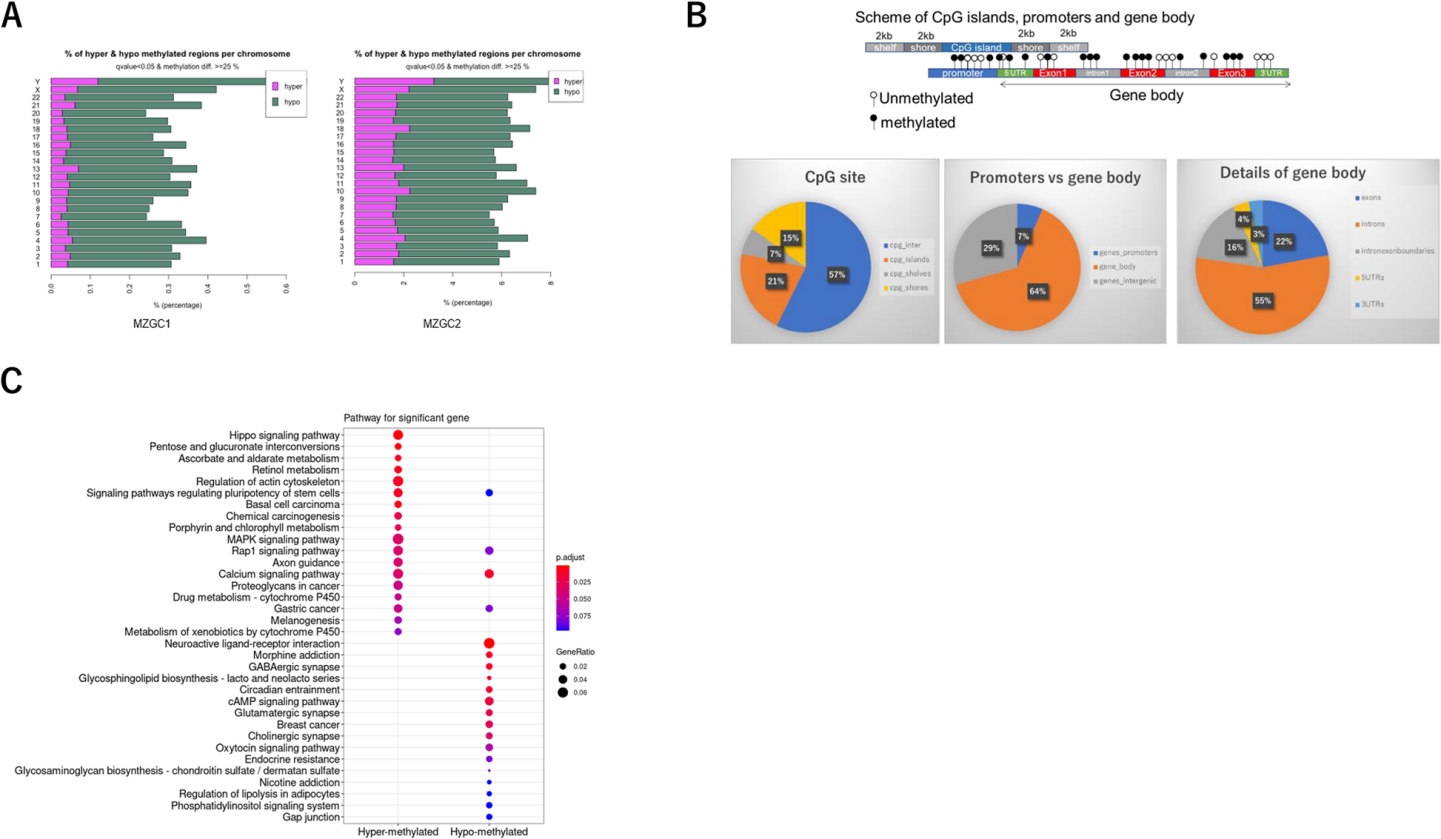
**A** Methionine depletion-induced global hypomethylation. CpGi methylation per chromosome (bar plot) from reduced representation of RRBS data indicating hypermethylated (pink) and hypomethylated (green) regions in the methionine depleted compared with control state in MZGC1. q Values < 0.05 and methylation difference ≥ 25%. **B** Scheme of CpGi, promoters, and gene body modified from Wang et al. [2] (upper) and distribution of DMRs (lower). DMRs of CpG site are mainly intergenic regions. Total amount of CpGi and shores of DMRs was 36%. Methylation of gene body, not just promoter, accounted for a large proportion. Breakdown shows that intron > exon > untranslated regions. **C** Pathways for significant genes analyzed by GeneDMRs. Significant gene ontology terms and pathways (adjusted *P* < 0.1) of the up- and down-regulated differentially expressed genes in the hyper/hypo-methylated categories by combining with the R package clusterProfiler

We tried to assess the relationship between transcriptome and DNA methylation status. Epigenetic analysis using RRBS revealed that depletion of methionine promoted genome wide hypomethylation in both promoter and gene body in many cases (Fig. 3A). We analyzed DMRs to identify DMR networks associated with affected pathways using the R package “GeneDMRs.” GeneDMRs can separately analyze the methylation levels within the gene, gene body, CpG island regions (CpGi) mainly localized in the promoter, and overlapping regions (Fig. 3B). Although there is some degree of variation between cell lines (Fig. 3A), analysis of raw data using GeneDMRs allowed statistically significant pathways to be picked up according to the degree of methylation. Pathway analysis of CpGi revealed that signaling pathways such as regulating pluripotency of stem cells, porphyrin and chlorophyll metabolism, retinol metabolism, regulation of actin cytoskeleton, and MAPK signaling pathway are hypermethylated (Fig. 3C) in the presence of methionine depletion. In contrast, regulation of lipolysis in adipocytes, circadian entrainment, neuroactive ligand-receptor interactions, glutamatergic synapse, cholinergic synapse, and phosphatidylinositol signaling system pathways were hypomethylated. CpGi analysis often classifies pathways as either hyper- or hypomethylated, but often overlap by gene body analysis (Supplementary Figure S3). Pathways classified as either by gene body analysis included focal adhesion and Kyoto Encyclopedia of Genes and Genomes glioma pathway which were hypomethylated (Supplementary Figure S3).

### Methionine depletion changed GIC pluripotency

**Fig. 4:**
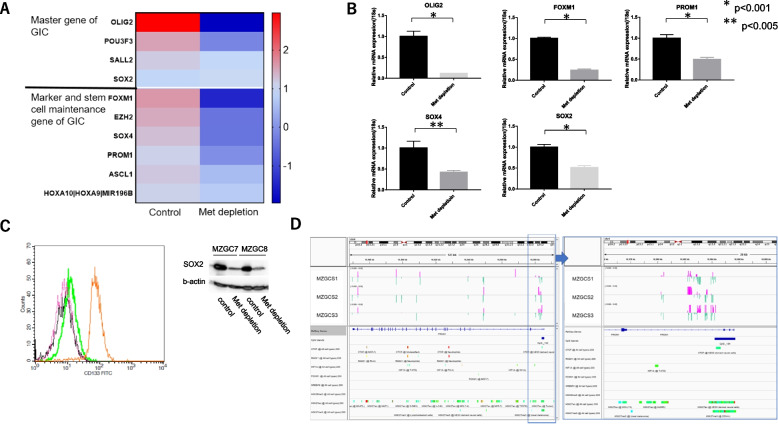
**A** Microarray results of top 10 master, maintenance, and marker genes changed by methionine depletion. Averages of the interested gene mRNA expression from the expression array are depicted using Prism7. **B** Validation with qPCR of master, maintenance, and marker genes of GICs. Methionine depletion down-regulated *OLIG2, SOX2, SOX4, FOXM1*, and *PROM1* mRNA gene expression in MZGC4. **C** FCM (left panel) and immunoblot analysis (right panel). Methionine depletion decreased markers of GICs CD133 and SOX2. Left panel: CD133 expression in cultured medium with (green, pink) / without (black) methionine in MZGC7. Methionine depletion for 5 days (Green) and 7 days (black) reduced CD133 expression to the same level of isotype control (pink). Orange overlay showed CD133 positive cells as positive control. Right panel: Methionine depletion for 5 days reduced SOX2 expression in MZGC7 and 8. Each experiment was performed in triplicate. (Full-length blots/gels are presented in Supplementary Figure S10A). **D** IGV showing gene locus of *PROM1* with DMRs of MZGC1, MZGC2, and MZGC3 along with gene map from Reference Sequence (RefSeq), CpGi, binding site of insulator, transcriptional factors, and histone markers from ChIP-seq database. DMRs by methionine depletion were localized in almost the same locus, where CpGi and gene body occur, in all cell lines. Close-up of the promoter region shows DMRs are localized around CpGi and shores (right). *FOXM1* binds to intron of *PROM1* gene body, whose histone mark is H3K27ac. Hypermethylated (pink) and hypomethylated (green) in the methionine depleted compared to control states are indicated. Hg19 used as reference genome

RRBS demonstrated the regulating pluripotency of stem cell pathways (Fig. 3C), so the expression of genes related to GIC pluripotency was investigated. Expression array analysis observed down-regulation of OLIG2 gene expression, one of the four core genes of GICs [3, 4], and down-regulation of *FOXM1*, *PROM1*, and other marker/maintenance genes of GICs (Fig. 4A). qPCR, FCM and immunoblotting experiments confirmed down-regulation of these markers (Fig. 4B and C). We investigated epigenetic changes of those genes. The CpGi in the promoter and genebody region of *PROM1* gene are mainly hypomethylated by the removal of Methionin, while the shore region is mainly hypermethylated. Interestingly, almost the same locus of the gene body was changed in three GICs (Fig. 4D).

### Methionine targets cholesterol metabolism: methionine removal and statin addition caused similar phenotypic changes in GICs

**Fig. 5:**
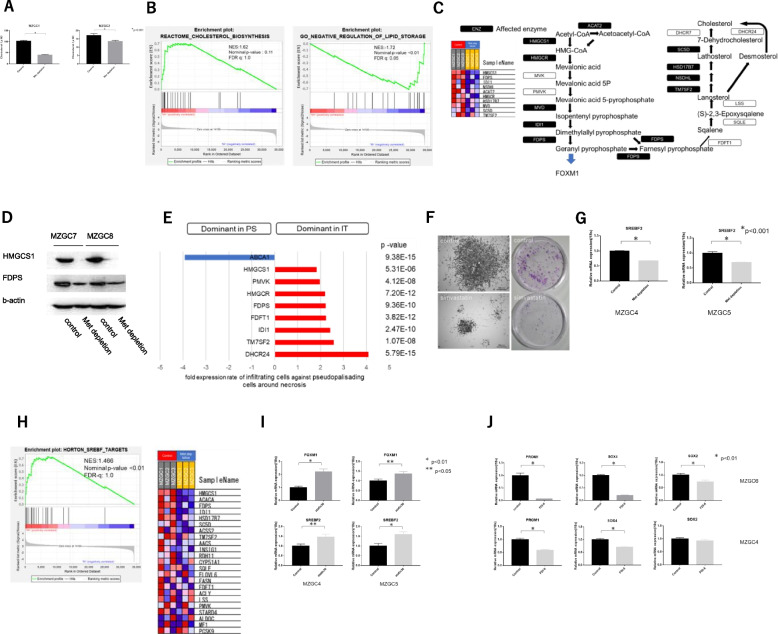
**A** Cholesterol concentration measured using 24(S)-hydroxycholesterol ELISA kit. Methionine depletion decreased cholesterol concentration in MZGC1 and MZGC2. **B** Enrichment analysis using GSEA showing that methionine depletion down-regulated the gene set of reactome cholesterol biosynthesis and up-regulated the gene set of gene ontology negative regulation of lipid storage. NES: normalized enrichment score, FDR-q: false discovery ratio. **C** Top10 genes from GSEA of affected enzyme related cholesterol biosynthesis shown in heat map and scheme of biosynthetic pathway of cholesterol (modified from Sitaula and Burris [5]). Selected genes are displayed in a black box with white text. **D** Immunoblot analysis of affected key enzymes related cholesterol biosynthesis. Methionine depletion for 5 days reduced HMGCS1 and FDPS. This experiment was performed in triplicate. (Full-length blots/gels are presented in Supplementary Figure S10B). **E** Differential expression of de novo biosynthesis and efflux of cholesterol hits with gene signature score in infiltrating cells against pseudopalisading cells around necrosis in all Ivy GAP samples. *ABCA1* (ATP-binding cassette subfamily A member 1), which acts as a cholesterol efflux pump in the cellular lipid removal pathway, is up-regulated in pseudopalisading cells around necrosis. Genes of cholesterol biosynthesis such as *HMGCS1*, *HMGCR*, and *FDPS* are up-regulated in infiltrating cells. **F** Simvastatin for 3 days decreased colony formation in GICs. High magnification (left) and low magnification (right). **G** qPCR showed down-regulated *SREBF2* mRNA expression by methionine depletion in MZGC4 and MZGC5. **H** Enrichment analysis by GSEA showed up-regulated Horton SREBF target gene set in an environment with sufficient methionine. **I** qPCR showed that short term statin administration up-regulated *FOXM1* mRNA expression along with up-regulated *SREBF2*. **J** qPCR showed FOXM1 inhibitor FDI-6 administration for 48 h down-regulated *SOX4, SOX2*, and *PROM1* mRNA expression

Metascape analysis (Fig. 2A) suggested that methionine depletion affects cholesterol metabolism, so a detailed study was conducted. First, we analyzed cholesterol consumption in GICs. Methionine depletion reduced cholesterol in GICs (Fig. 5A). GSEA showed that methionine depletion reduced cholesterol biosynthesis and increased cholesterol excretion pathways (Fig. 5B). The 10 enzymes of cholesterol biosynthesis (*HMGCS1, FDPS, IDI1, NSDHL, ACAT2, HMGCR, HSD17B7, MVD, SC5D,* and *TM7SF2*) showing greatest decreases are shown in the schema (Fig. 5C) We also confirmed reduced protein level by methionine depletion (Fig. 5D).

Next, we compared the expression levels of genes of cholesterol metabolism within the tumor mass (perinecrotic pseudopalisading area) with the infiltrating area using Ivy GAP database. The genes related to de novo synthesis of cholesterol were predominantly up-regulated in infiltrating tumor cells compared with tumor cells in the perinecrotic pseudopalisading area (Fig. 5E, Supplementary Figure S4). However, *ABCA1*, which acts as the efflux pump of cholesterol, showed the opposite distribution. Simvastatin was added to assess whether chemical inhibition of *HMGCR* had same effect with methionine depletion. Simvastatin decreased colony formation, showing a very similar phenotypic change to methionine depletion (Fig. 5F, Supplementary Figure S5) and down-regulation of the stem cell markers *FOXM1, PROM1, OLIG2,* and *SOX2* (Supplementary Figure S6A, B and S10C). These results indicated that methionine metabolism is deeply involved in the survival, proliferation, and pluripotency of GICs through cholesterol metabolism.

IGV investigation of the methylation status of each gene found no significant change in gene body or CpGi as in *HMGCS1* (Fig. S7A). We think that the cholesterol metabolic genes might be mainly regulated by another mechanism. We analyzed the transcription factor candidates and insulator binding sites that are likely to bind to the promoter region from the ChIP-seq database. CpGi in the promoter of *HMGCS1* had no DMRs, but *CTCF* and *SREBF2* binding are reported (Fig. S7A). Since *SREBF2* is known to be an important regulator of cholesterol metabolism, we checked the expression of *SREBF2*. *SREBF2* was down-regulated by methionine depletion (Fig. 5G). Moreover, we found multiple genes in the Horton SREBF target gene set were down-regulated by methionine depletion (Fig. 5H). Among the top 10 affected genes related to cholesterol biosynthesis (Fig. 5C), *HMGCS1, FDPS, IDI1, SC5D, TM7SF2,* and *HSD17B7* are *SREBF* target genes. Therefore, we thought that *SREBF2* was one of the key molecules linking methionine and cholesterol metabolism. To assess the methylation status of *SREBF2*, we applied the data of RRBS to IGV. DMRs were mainly found in the gene body including 3’-untranslated region (Fig. S7B).

We then considered whether cholesterol metabolism is related to the pluripotency of GICs. *FOXM1*, a maintenance gene of GICs, is also associated with cholesterol metabolism [6]. Thus, we investigated the relationship between cholesterol metabolism and *FOXM1*. *FOXM1* was down-regulated with *SREBF2* by methionine depletion (Figs. 4A, B, and 5G). *SREBF2* increased transiently in the short time after statin administration with feedback that cholesterol production was reduced, but then decreased in the longer term (Fig. 5I). *FOXM1* was up-regulated together with up-regulated *SREBF2* (Fig. 5 I). IGV showed many hypomethylated DMRs in the gene body and CpGi in the promoter of *FOXM1*, which includes the *CTCF* binding site (Fig. S7C). Interestingly, this site has been reported to bind with *SREBF2* (Fig. S7C). To assess whether *FOXM1* regulates the core/marker genes of GICs, we used FDI-6 as a chemical inhibitor of *FOXM1*. FDI-6 down-regulated *SOX4*, *SOX2,* and *PROM1* (Fig. 5J). This observation suggests that methionine metabolism altered the pluripotency of GICs through the cholesterol metabolism to the *FOXM1* axis.

### Methionine also targets protein synthesis

**Fig. 6:**
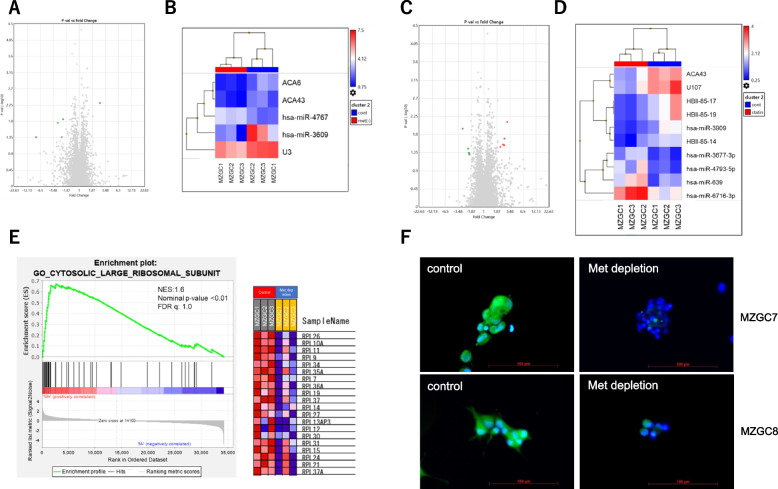
**A**, **C** Volcano plot showing the miRNA distribution in control versus methionine-depleted groups (**A**) and in control versus statin-treated groups (**C**). Up- (red color-positive fold change) and down-regulation (green color-negative fold change) in the treated group compared to the control group are indicated. Hierarchical clustering of the differentially expressed miRNAs (*P* value ˂ 0.05 and change ≥ 2.0 fold or ≤  − 2.0 fold) in the treatment group compared with the control group showed a clear distinction between control and treatment groups in miRNA including small noncoding RNAs. **B**, **D** Treatments are methionine depletion (**B**) and simvastatin administration for 48 h (**D**). Color scale indicates the relative expression of miRNAs: red shows higher expression and blue lower expression. **E** GSEA of differentially expressed genes. The enrichment profiles for “gene ontology cytosolic large ribosomal subunit” of three cell lines are shown. Heat map of top 20 genes from GSEA are shown on the right. Red column corresponds to upregulated genes and blue column corresponds to downregulated genes. NES: normalized enrichment score, FDR-q: false discovery ratio. **F** Methionine depletion reduced protein synthesis. We can detect the 5 FAM-Azide labeled protein in only cultured with methionine contained medium

*hsa-miR-33A* has a DNA locus presenting many DMRs, adjacent to the *SREBF2* locus (Fig. S7B), and is known to cause post-translational modifications of genes involved in cholesterol metabolism [7], so we analyzed non-coding RNAs using miRNA arrays. Exhaustive analysis of methionine depletion and statin administration identified one common *SNORA17B (ACA43)*, but not *hsa-miR-33A* (Fig. 6A–D). *ACA43* is predicted to induce pseudouridylation of U4938 of a 28S rRNA. Most pseudouridylation sites are located within functionally important ribosomal domains, which may affect the functional features of the ribosome. We thought that another common target of methionine and cholesterol metabolism might be a ribosomal protein. Entry of the mRNA expression data into GSEA using ontology gene sets found that gene ontology cytosolic large ribosomal subunit gene expression was extremely upregulated in an environment with adequate methionine (Fig. 6E). Identified RPL genes such as *RPL26, RPL10A, RPL11, RPL9,* and *RPL34* are all constituent proteins of a large subunit of 28S, with the function to bind tRNA during the synthesis of polypeptides. We also confirmed that methionine depletion reduces protein synthesis with OPP assay (Fig. 6F). Since epitranscriptome modifications such as pseudourinalization include methylation, we searched for genes involved in epitranscriptome methylation affected by methionine metabolic reorganization. The transcriptome Venn diagram showed methyltransferase-like protein 16 (*METTL16*), which is related to *MAT2A*, and methyltransferase-like protein 20 (*METTL20*) were predominantly up-regulated by methionine depletion (Table 1).

**Table 1:**
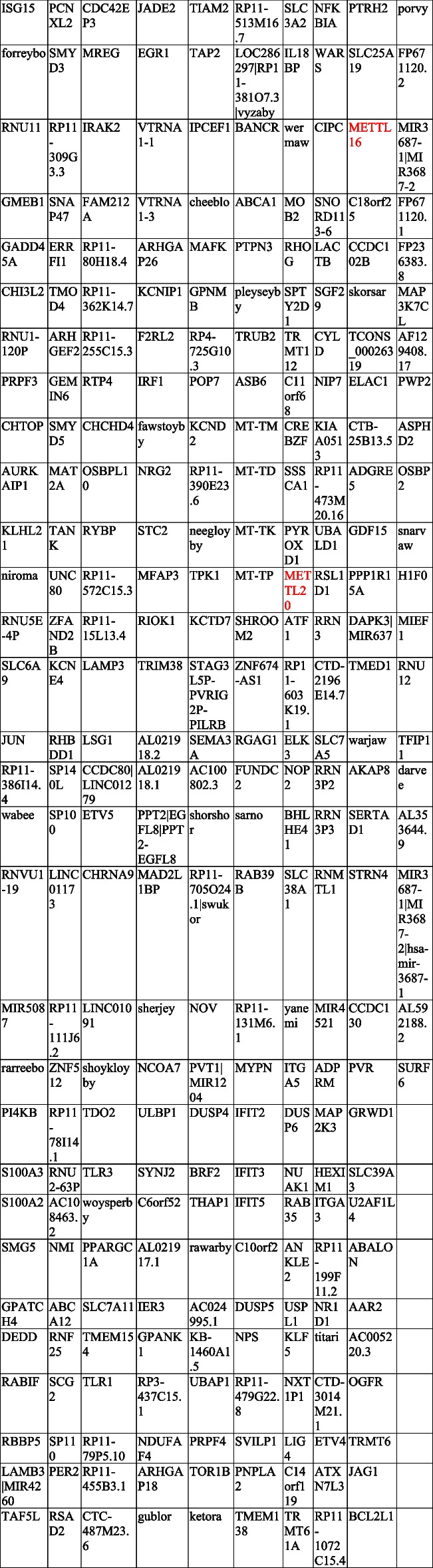
Genes significantly up-regulated with change cut-off at 2.0 fold and *P* value cut-off at 0.05 in all MZGC1, MZGC2, and MZGC3 during methionine depletion

*METTL16* and *METTL20* are depicted in red

## Discussion

SAM is the key molecule of transmethylation reactions and polyamine biosynthesis using methionine [8]. The biosynthesis of SAM is catalyzed by three major MATs, I, II, and III [9]. MATI and MATIII are encoded by *MAT1A*. MATII consists of an α2 catalytic subunit encoded by *MAT2A* and the β-regulatory subunit encoded by *MAT2B*. MATII functions as a transcriptional corepressor in the oxidative stress response, forming a SAM-integrating transcription regulation module that influences histone methyltransferase activity [10, 11]. We demonstrated that methionine depletion caused decreased SAM and increased *MAT2A* and *MAT2B* in time-dependent manner. As a result, some genes are hypomethylated and others are hypermethylated, although the genome as a whole tends towards hypomethylation. Analysis of signaling pathways to determine what groups of genes are altered also shows a clear distinction between hyper- and hypo- methylation for each pathway. We speculate that this is probably where the reorganisation takes place, with the intention of gene expression to escape methionine depletion and allow the cell to survive. Although we did not search for the mechanism in this study, we believe that a search for the methylation status of histones and gene expression will be necessary in the future, as there are reports that SAM metabolism affects genome-wide histone methylation and is involved in the maintenance of pluripotent stem cells [12]. Since we expected decreased SAM to result in genomic hypomethylation of the promoter and gene body of *MAT2A*, we entered the RRBS data into IGV. Epigenetic methylation status of *MAT2A* and *MAT2B* were not so changed in spite of the marked increase in mRNA gene expression (Fig. S8). Thus, we searched for genes that affect the epi-transcriptome modulation using microarray results to understand the mechanism that causes the increase in key molecule *MAT2A* under the methionine-depleted state. We found two up-regulated N6-adenosine-methyltransferases, *METTL16* and *METTL20*, which are involved in the post-transcriptional methylation of internal adenosine residues in eukaryotic mRNAs to form N6-methyladenosine). In agreement with our data, increased *METTL16* occupancy on *hp1* increased *MAT2A* splicing under SAM-limiting conditions [13]. Cells induce *MAT2A* expression by enhanced splicing of a retained intron. Interestingly, *METTL16* also targets *ACA43*, one of the key factors shared by cholesterol and methionine metabolism, as discussed below [14].

Another up-regulated N6-methyladenosine methyltransferase, *METTL20* (also known as electron transfer flavoprotein subunit beta lysine methyltransferase), is a mitochondrial lysine protein methylase that modifies Lys-199 and Lys-202 of the electron transfer flavoprotein subunit beta, which is a mobile electron carrier coupling mitochondrial fatty acid oxidation to respiration [15, 16]. Investigation using the knockout mouse showed *METTL20* is a positive regulator of beta-oxidation. Beta-oxidation is known as a metabolic node involved in glioma-genesis [17], and also provides an alternate source of ATP in nutrient unfavorable conditions. Our observation about *METTL20* may represent the steps to obtain ATP.

Acetyl CoA is an important intermediate in the metabolism of lipids for energy storage and supply, from which fatty acids and cholesterol are synthesized. As mentioned above, beta-oxidation of fatty acids may be affected by methionine deprivation, but the metabolism of cholesterol was also greatly affected. Methionine depletion downregulates cholesterol biosynthesis and increases excretion. Furthermore, methionine depletion and statin administration showed similar phenotypic changes in GICs. These observations suggest that cholesterol metabolism is necessary to maintain the pluripotency of GICs. Biosynthesis of cholesterol is tightly regulated by feedback pathways because cholesterol is critical for cell growth and function. Cholesterol is important as a component of the plasma membrane and lipid rafts, and as a precursor for steroid hormones, bile acids, and vitamin D [18, 19]. Other organs are more dependent on cholesterol supply from the bloodstream, whereas the brain obtains cholesterol exclusively from de novo synthesis, which involves the mevalonate and Bloch and Kandutsch-Russell pathways [20–22].

Within a tumor mass, imbalances in methionine concentration as well as oxygen concentration should exist. Using the Ivy GAP database, we compared the expression of genes involved in cholesterogenesis in the pseudopalisading cells and infiltrating tumor portions [23]. Our finding that cholesterogenesis was higher in the infiltrating tumor than in the pseudopalisading cell areas was consistent with the results of in vitro experiments. De novo synthesis is mediated by the transcriptional activity of sterol regulatory element binding proteins (SREBPs), which promote the transcription of enzymes involved in the cholesterol and fatty acid biosynthetic pathways, such as *FDPS* and *HMGCR*. When cholesterol levels fall below homeostatic levels, SREBP cleavage activating protein is isolated from insulin-inducible genes (*INSIG1, INSIG2*) resident in the endoplasmic reticulum [18, 24]. The SREBP is then transported to the Golgi, where the active site is cleaved and activated as a transcription factor [25]. Among the three types (SREBP1a, SREBP1c, and SREBP2), SREBP2, which is encoded by the *SREBF2* gene, mainly regulates genes involved with cholesterol biosynthesis. *SREBF2* is upregulated with statin treatment by feedback but down-regulated by methionine depletion (Fig. 5G and J). Such downregulation of *SREBF2* under methionine depletion is thought to be the main cause of the downregulation of SREBF target genes of cholesterol biosynthesis.

Other groups have evaluated the effects of SREBPs on glioblastoma multiform development as a key transcription factor in the regulation of sterol homeostasis [26, 27]. Under hypoxia and serum deprivation conditions, SREBP is upregulated to maintain the expression of fatty acid and cholesterol biosynthesis genes in glioblastoma multiform cells, and inhibition of SREBP activity under hypoxia results in the death of glioblastoma multiform cells [18, 27]. So how does *SREBF2* decrease under methionine depletion? We cannot determine the detailed mechanism, but the gene body of *SREBF2*, especially the 3’ side including the hsa-miR-33A locus, is dramatically hypomethylated, and this might be the cause of down-regulation of *SREBF2*. The *hsa-miR-33A* is also known as regulator of cholesterol efflux through *ABCA1* gene expression [28].

To identify which miRNA contributes to the association between cholesterol metabolism and methionine depletion, we decided to explore the commonalities of changes in the miRNA array with methionine depletion and statin administration. Unexpectedly, only *ACA43* but not *hsa-miR-33A* was downregulated in both arrays. *ACA43* belongs to the H/ACA box class of small nucleolar RNAs based on the predicted hairpin-hinge-hairpin-tail structure and a conserved H/ACA-box motif. *ACA43* is predicted to induce pseudouridylation of U4938, a 28S rRNA. Pseudouridylation is the isomerization of the nucleoside uridine to a different isomer, pseudouridine. Interestingly, *ACA43* was originally cloned from HeLa cells [29] and is associated with the GAR1 protein, one of the components of the telomerase complex [30]. Taken together, these reports and our findings may indicate that cholesterol metabolism and methionine metabolism are also involved in cell immortalization via *ACA43*. At least, cholesterol and methionine metabolism altered the translation of mRNA through downregulated *ACA43*. Translation was also suppressed through down-regulation of the large subunit of ribosomal protein 28S by methionine depletion (Fig. 6E and F). The novel anticancer drug rocaglamide A, which inhibits translation by binding to the translation initiation factor eIF4A, inhibits the proliferation of target cancer cells, suggesting that translation inhibition due to methionine depletion may have inhibited the growth of GICs [31, 32].

The final question is whether cholesterol metabolism is related to the apoptosis and pluripotency of GICs. Pharmacological inhibitors acting downstream of the mevalonate pathway induce cell death in glioma and pluripotent stem cells [33, 34]. Inhibition of *FDPS* by pharmacological inhibitors and small interfering RNA alters the self-renewal of glioma stem cells [35]. *HMGCS1* contributes to gastric cancer progression through activating *Oct4* and *SOX2* promoters [36]. These observations support our findings that methionine depletion promotes down-regulation of *SOX2, OLIG2, PROM1,* and *FOXM1* along with down-regulation of cholesterol de novo biosynthesis. *FOXM1* had a positive correlation with mevalonate pathway-related genes including *HMGCR* and *SREBP2* in patients with hepatocellular carcinoma. Moreover, knockdown of *HMGCR* reduced *FOXM1* expression in hepatocellular carcinoma [6]. We observed *FOXM1* was increased by statin administration for a short period and decreased by methionine depletion along with *SREBF2*. Moreover, IGV showed first hypo-methylated gene body and CpGi of the *FOXM1* promoter by methionine depletion and the *SREBF2* binding site in CpGi of the *FOXM1* promoter. These findings strongly suggest that methionine down-regulates *FOXM1* through *SREBF2*. Since *FDPS* and *HMGCS1* are targets of *SREBF2, SREBF2* might be a key molecule-connecting cholesterol metabolism and pluripotency of stem cells. Finally, we found that the chemical inhibitor of *FOXM1* downregulates core and marker genes of GICs. Taken together, we thought *SREBF2-FOXM1* are important regulators of the core/marker genes in GICs (Fig. S9).

In conclusion, we found that methionine metabolism is closely related to cholesterol metabolism and ribosomal function, resulting in alteration of pluripotency, proliferation, and avoidance of apoptosis in GICs. 

## Supplementary Information

**Additional file 1:** **Supplementary Table S1.** RRBS libraries and Sequence data generated in this study.

**Additional file 2:** **Supplementary Table S2.** Primers sequence used in this study.

**Additional file 3:** **Supplementary Figure S1.** **A** Fluorescence-activated cell sorter analysis to detect cell cycle distribution. Propidium iodide (PI) staining assay using a Cell Cycle Phase Determination Kit was performed on MZGC6 cells. Cell cycle analysis showed that methionine depletion for 3 days increased sub-G1 and decreased S, G2M populations. **B** Dye exclusion test showed that methionine depletion (for 5 and 7 days) and statin treatment (for 1 and 2 days) increased dead cell rate in MZGC7. **C** Fluorescence-activated cell sorter analysis using annexin-V kit showed that methionine depletion induced apoptotic cell death. **Supplementary Figure S2.** Microarray analysis using Metascape. Heat map showing commonly altered pathways in the control and methionine-depleted states of three GICs (MZGC1, MZGC2, and MZGC3). Statistically enriched terms (gene ontology/Kyoto Encyclopedia of Genes and Genomes terms, canonical pathways, hallmark gene sets) are given with their *P* value. The 100 families of pathways with the lowest -log10 adjusted *P* value are depicted. **Supplementary Figure S3.** Significant gene ontology terms and pathways for significant genes analyzed with gene body methylation status by GeneDMRs. Significant gene ontology terms and pathways (adjusted *P* < 0.1) of the up- and down-regulated differentially expressed genes in the hyper/hypo-methylated categories by combining with the R package clusterProfiler. **Supplementary Figure S4.** Z-score of gene expression from Ivy GAP. Gene expression related de novo synthesis of cholesterol was upregulated in infiltrating tumor cells compared with pseudopalisading cells around necrosis. In contrast, the gene expression pattern related to efflux of cholesterol was exactly the opposite. **Supplementary Figure S6.** **A** qPCR of stem cell markers in MZGC4 and MZGC5. Statin treatment for 120 h down-regulated mRNA expression of* FOXM1, PROM1, OLIG2*,and *SOX2*. **B** Immunoblotting analysis showed that statin treatment for 2 days reduced SOX2protein level as well as methionine depletion for 7 days in MZGC7 and 8. (Full-length blots/gels are presented in **Supplementary Figure S10C**). **Supplementary Figure S7.** **A** IGV showed few DMRs in promoter and gene body of HMGCS1. CTCF/RAD21, HIF1a, and SREBF2 binding site are reported in the CpGi of promoter, where histone marks are H3K27Ac and H3K27me3. **B** IGV showed the DMRs of SREBF2. DMRs of promoter werenot found, but the binding of insulator CTCF and HIF1a were identified. DMRs were mainly localized in the gene body in the 3' side of the gene body, especially near the hsa-miR-33A locus. **C** IGV showed DMRs of FOXM1. Many DMRsare found in both gene body and promoter. CTCF, HIF1a, and SREBF2 are reported to bind to the lesion of DMRs in promoter, and H3K27ac may be a histone mark. **Supplementary Figure S8.** IGV showed few DMRs in the promoter and genebody of MAT2A and MAT2B. **Supplementary Figure S9.** Graphical summary. Interaction of the major factors and events by reorganization of methionine metabolism. **Supplementary Figure S10.** Full-length blots/gels and cropped lesions of Fig. 4C (**A**), Fig. 5D (**B**) and **Supplementary Figure S6B** (**C**) were indicated.

**Additional file 4.** Supplementary methods.

**Additional file 5:** Supplementary video **Figure S5**.

## Data Availability

The raw sequencing data have been deposited to the DNA Data Bank of Sequence Read Archive under the BioProject PRJDB12471 (https://www.ebi.ac.uk/ena/browser/view/PRJDB12471) with run accession numbers DRR322659, DRR322660, DRR322661, DRR322662, DRR322663 and DRR322664. (Supplementary Table S1) The data discussed in this publication are also available in GEA under the accession number E-GEAD-458.

## References

[1] Diplas BH, He X, Brosnan-Cashman JA, Liu H, Chen LH, Wang Z, Moure CJ, Killela PJ, Loriaux DB, Lipp ES (2018). The genomic landscape of TERT promoter wildtype-IDH wildtype glioblastoma. Nat Commun.

[2] Wang X, Hao D, Kadarmideen HN (2021). GeneDMRs: An R Package for Gene-Based Differentially Methylated Regions Analysis. J Comput Biol.

[3] Fiscon G, Conte F, Paci P (2018). SWIM tool application to expression data of glioblastoma stem-like cell lines, corresponding primary tumors and conventional glioma cell lines. BMC Bioinformatics.

[4] Lathia JD, Mack SC, Mulkearns-Hubert EE, Valentim CL, Rich JN (2015). Cancer stem cells in glioblastoma. Genes Dev.

[5] Sitaula S (2016). TPB: Cholesterol and Other Steroids: Academic Press.

[6] Ogura S, Yoshida Y, Kurahashi T, Egawa M, Furuta K, Kiso S, Kamada Y, Hikita H, Eguchi H, Ogita H (2018). Targeting the mevalonate pathway is a novel therapeutic approach to inhibit oncogenic FoxM1 transcription factor in human hepatocellular carcinoma. Oncotarget.

[7] Ono K, Horie T, Nishino T, Baba O, Kuwabara Y, Yokode M, Kita T, Kimura T (2015). MicroRNA-33a/b in lipid metabolism - novel "thrifty" models. Circ J.

[8] Shiraki N, Shiraki Y, Tsuyama T, Obata F, Miura M, Nagae G, Aburatani H, Kume K, Endo F, Kume S (2014). Methionine metabolism regulates maintenance and differentiation of human pluripotent stem cells. Cell Metab.

[9] Halim AB, LeGros L, Geller A, Kotb M (1999). Expression and functional interaction of the catalytic and regulatory subunits of human methionine adenosyltransferase in mammalian cells. J Biol Chem.

[10] Goll MG, Bestor TH (2005). Eukaryotic cytosine methyltransferases. Annu Rev Biochem.

[11] Lu SC, Mato JM (2008). S-Adenosylmethionine in cell growth, apoptosis and liver cancer. J Gastroenterol Hepatol.

[12] Shyh-Chang N, Locasale JW, Lyssiotis CA, Zheng Y, Teo RY, Ratanasirintrawoot S, Zhang J, Onder T, Unternaehrer JJ, Zhu H (2013). Influence of threonine metabolism on S-adenosylmethionine and histone methylation. Science.

[13] Pendleton KE, Chen B, Liu K, Hunter OV, Xie Y, Tu BP, Conrad NK (2017). The U6 snRNA m(6)A Methyltransferase METTL16 Regulates SAM Synthetase Intron Retention. Cell.

[14] Warda AS, Kretschmer J, Hackert P, Lenz C, Urlaub H, Hobartner C, Sloan KE, Bohnsack MT (2017). Human METTL16 is a N(6)-methyladenosine (m(6)A) methyltransferase that targets pre-mRNAs and various non-coding RNAs. EMBO Rep.

[15] Rhein VF, Carroll J, He J, Ding S, Fearnley IM, Walker JE (2014). Human METTL20 methylates lysine residues adjacent to the recognition loop of the electron transfer flavoprotein in mitochondria. J Biol Chem.

[16] Shimazu T, Furuse T, Balan S, Yamada I, Okuno S, Iwanari H, Suzuki T, Hamakubo T, Dohmae N, Yoshikawa T (2018). Role of METTL20 in regulating beta-oxidation and heat production in mice under fasting or ketogenic conditions. Sci Rep.

[17] Kant S, Kesarwani P, Prabhu A, Graham SF, Buelow KL, Nakano I, Chinnaiyan P (2020). Enhanced fatty acid oxidation provides glioblastoma cells metabolic plasticity to accommodate to its dynamic nutrient microenvironment. Cell Death Dis.

[18] Ahmad F, Sun Q, Patel D, Stommel JM (2019). Cholesterol Metabolism: A Potential Therapeutic Target in Glioblastoma. Cancers (Basel).

[19] Janowski BA, Willy PJ, Devi TR, Falck JR, Mangelsdorf DJ (1996). An oxysterol signalling pathway mediated by the nuclear receptor LXR alpha. Nature.

[20] Bloch K (1965). The biological synthesis of cholesterol. Science.

[21] Goldstein JL, Brown MS (1990). Regulation of the mevalonate pathway. Nature.

[22] Kandutsch AA, Russell AE (1960). Preputial gland tumor sterols. 3. A metabolic pathway from lanosterol to cholesterol. J Biol Chem.

[23] Puchalski RB, Shah N, Miller J, Dalley R, Nomura SR, Yoon JG, Smith KA, Lankerovich M, Bertagnolli D, Bickley K (2018). An anatomic transcriptional atlas of human glioblastoma. Science.

[24] Adams CM, Reitz J, De Brabander JK, Feramisco JD, Li L, Brown MS, Goldstein JL (2004). Cholesterol and 25-hydroxycholesterol inhibit activation of SREBPs by different mechanisms, both involving SCAP and Insigs. J Biol Chem.

[25] Sakai J, Duncan EA, Rawson RB, Hua X, Brown MS, Goldstein JL (1996). Sterol-regulated release of SREBP-2 from cell membranes requires two sequential cleavages, one within a transmembrane segment. Cell.

[26] Geng F, Cheng X, Wu X, Yoo JY, Cheng C, Guo JY, Mo X, Ru P, Hurwitz B, Kim SH (2016). Inhibition of SOAT1 Suppresses Glioblastoma Growth via Blocking SREBP-1-Mediated Lipogenesis. Clin Cancer Res.

[27] Lewis CA, Brault C, Peck B, Bensaad K, Griffiths B, Mitter R, Chakravarty P, East P, Dankworth B, Alibhai D (2015). SREBP maintains lipid biosynthesis and viability of cancer cells under lipid- and oxygen-deprived conditions and defines a gene signature associated with poor survival in glioblastoma multiforme. Oncogene.

[28] Horie T, Ono K, Horiguchi M, Nishi H, Nakamura T, Nagao K, Kinoshita M, Kuwabara Y, Marusawa H, Iwanaga Y (2010). MicroRNA-33 encoded by an intron of sterol regulatory element-binding protein 2 (Srebp2) regulates HDL in vivo. Proc Natl Acad Sci U S A.

[29] Kiss AM, Jady BE, Bertrand E, Kiss T (2004). Human box H/ACA pseudouridylation guide RNA machinery. Mol Cell Biol.

[30] Jafri MA, Ansari SA, Alqahtani MH, Shay JW (2016). Roles of telomeres and telomerase in cancer, and advances in telomerase-targeted therapies. Genome Med.

[31] Chen M, Asanuma M, Takahashi M, Shichino Y, Mito M, Fujiwara K, Saito H, Floor SN, Ingolia NT, Sodeoka M (2021). Dual targeting of DDX3 and eIF4A by the translation inhibitor rocaglamide A. Cell Chem Biol.

[32] Schulz G, Victoria C, Kirschning A, Steinmann E (2021). Rocaglamide and silvestrol: a long story from anti-tumor to anti-coronavirus compounds. Nat Prod Rep.

[33] Kambach DM, Halim AS, Cauer AG, Sun Q, Tristan CA, Celiku O, Kesarwala AH, Shankavaram U, Batchelor E, Stommel JM (2017). Disabled cell density sensing leads to dysregulated cholesterol synthesis in glioblastoma. Oncotarget.

[34] Nakashima Y, Miyagi-Shiohira C, Noguchi H, Omasa T (2018). Atorvastatin Inhibits the HIF1alpha-PPAR Axis, Which Is Essential for Maintaining the Function of Human Induced Pluripotent Stem Cells. Mol Ther.

[35] Kim HY, Kim DK, Bae SH, Gwak H, Jeon JH, Kim JK, Lee BI, You HJ, Shin DH, Kim YH (2018). Farnesyl diphosphate synthase is important for the maintenance of glioblastoma stemness. Exp Mol Med.

[36] Wang IH, Huang TT, Chen JL, Chu LW, Ping YH, Hsu KW, Huang KH, Fang WL, Lee HC, Chen CF (2020). Mevalonate Pathway Enzyme HMGCS1 Contributes to Gastric Cancer Progression. Cancers (Basel).

