# Supplementary material for: Methionine regulates self-renewal, pluripotency, and cell death of GIC through cholesterol—rRNA axis
Source: BMC Cancer. 2022 Dec 23;22:1351. doi: 10.1186/s12885-022-10280-5 (PMC9789638; doi:10.1186/s12885-022-10280-5)
Supplement: Supplementary file 1 — Additional file 1: Supplementary Table S1. RRBS libraries and Sequence data generated in this study. [file 12885_2022_10280_MOESM1_ESM.docx]

**Supplementary Table S1.** RRBS libraries and Sequence data generated in this study

| Library  name | Cell  name | sample type | Library type | accession  number | sequencing  platform | read length  (bp) | number of reads | sequenced  bases | Clean Q30 Bases Rate(%) |
| --- | --- | --- | --- | --- | --- | --- | --- | --- | --- |
| MZGC1c | MZGC1 | Control media | pair-end | DRR322659 | HiSeqX | 150 | 106,555,558 | 15,983,333,700 | 83.62 |
| MZGC1s | MZGC1 | Methyonine depleted media | pair-end | DRR322660 | HiSeqX | 150 | 109,768,722 | 16,465,308,300 | 84.07 |
| MZGC2c | MZGC2 | Control media | pair-end | DRR322661 | HiSeqX | 150 | 138,833,106 | 20,824,965,900 | 84.95 |
| MZGC2s | MZGC2 | Methyonine depleted media | pair-end | DRR322662 | HiSeqX | 150 | 104,742,544 | 15,711,381,600 | 85.9 |
| MZGC3c | MZGC3 | Control media | pair-end | DRR322663 | HiSeqX | 150 | 86,377,632 | 12,956,644,800 | 84.53 |
| MZGC3s | MZGC3 | Methyonine depleted media | pair-end | DRR322664 | HiSeqX | 150 | 115,347,352 | 17,302,102,800 | 84.58 |
