# Supplementary material for: Methionine regulates self-renewal, pluripotency, and cell death of GIC through cholesterol—rRNA axis
Source: BMC Cancer. 2022 Dec 23;22:1351. doi: 10.1186/s12885-022-10280-5 (PMC9789638; doi:10.1186/s12885-022-10280-5)
Supplement: Supplementary file 2 — Additional file 2: Supplementary Table S2. Primers sequence used in this study. [file 12885_2022_10280_MOESM2_ESM.docx]

Supplementary Table S2 Primers sequence used in this study

|  | Forward (5' to 3') | Reverse (5' to 3') |
| --- | --- | --- |
| MAT2A | CCACGAGGCGTTCATCGAGG | AAGTCTTGTAGTCAAAACCT |
| MAT2B | TGGGGAGCACTTGAAAGAG | CTTAGCGGCAACATGGG |
| OLIG2 | CGCCAGAGCCCGATGACCTT | GACACGGTGCCCCCAGTGAA |
| FOXM1 | ACTTTAAGCACATTGCCAAGC | CGTGCAGGGAAAGGTTGT |
| PROM1 | CAGAGTACAACGCCAAACCA | AAATCACGATGAGGGTCAGC |
| SOX4 | AGCGACAAGATCCCTTTCATTC | CGTTGCCGGACTTCACCTT |
| SOX2 | GCACATGAACGGCTGGAGCAACG | TGCTGCGAGTAGGACATGCTGTAGG |
| SREBF2 | CCGGGCGCAACGCAAAC | CGCCCATGACACCCGACAA |
| 18S | GGCGCCCCCTCGATGCTCTTAG | GCTCGGGCCTGCTTTGAACACTCT |
