# Supplementary material for: Methionine regulates self-renewal, pluripotency, and cell death of GIC through cholesterol—rRNA axis
Source: BMC Cancer. 2022 Dec 23;22:1351. doi: 10.1186/s12885-022-10280-5 (PMC9789638; doi:10.1186/s12885-022-10280-5)
Supplement: Supplementary file 4 — Additional file 4. Supplementary methods. [file 12885_2022_10280_MOESM4_ESM.docx]

**Supplementary methods**

Protein synthesis assay

1x10^6^ cells were seeded and cultured for 5 days in CSC media with or without methionine. Samples were further processed according to the manufacturer's instructions using Protein Synthesis Assay Kit (601100, Cayman Chemical, Ann Arbor, MI, USA). In brief, cells were resuspended with OPP Working Solution for 30 min at 37 °C, washed and stained with the 5 FAM-Azide Staining Solution and DAPI. Cells were observed with Zeiss fluorescence microscopy Axio imager A2 (New York, USA).

Western Blotting

Immunoblotting was performed using standard protocol. In brief, cells were lysed with RIPA buffer, then sonicated. Total cell lysates were resolved in 12.5% acrylamide gels and transferred to a PVDF membrane (Merck Millipore, Germany). The membrane was incubated with 5% nonfat dry milk as blocking solution for 1h and reacted with indicated primary antibodies overnight at 4 degree. The membrane were then incubated for 1 h with HRP-conjugated secondary antibodies (DAKO, 1: 4000 dilution) in TBST at room temperature. The immunocomplexes were detected using chemiluminescent reagent. Used primary antibodies were as follows: SOX2 (#3579 ,1:1000 dilution), HMGCS1(#36877 , 1:1000 dilution) purchased from Cell Signaling Technology (CST) Japan, FDPS(ab153805, 1:1000 dilution) purchased from Abcam. Beta-actin (A5441, 1:5000 dilution) purchased from Sigma-Aldrich. Each experiment was performed in triplicate.
